# Decolonization of Global Health in Haiti: A Call for Equity, Partnerships, Scholarship, and Informed Action

**DOI:** 10.9745/GHSP-D-22-00298

**Published:** 2023-06-21

**Authors:** Dominique Guillaume, Regine Thermy, Candice Sternberg, Judith Seme, Tyra Montour, Balkys Bivins, Pepita Jean, Juanita Barnett, Guerda Nicolas

**Affiliations:** a Center for Infectious Disease and Nursing Innovation, Johns Hopkins University, Baltimore, MD, USA.; b Jhpiego, Baltimore, MD, USA.; c School of Education and Human Development, University of Miami, Miami, FL, USA.; d Division of Infectious Disease, Department of Medicine, University of Miami, Miami, FL, USA.; e Department of Nursing, West Coast University, Miami, FL, USA.; f Department of Health Behavior, School of Public Health, Texas A&M University, College Station, TX, USA.; g School of Nursing and Health Studies, University of Miami, Miami, FL, USA.; h Transplantation and Cellular Therapy, University of Miami Sylvester Cancer Center, Miami, FL, USA.; i Counselor Education Program, Alabama State University, Montgomery, AL, USA.; j Department of Educational and Psychological Studies, School of Education and Human Development, University of Miami, Miami, FL, USA.

## Abstract

Haiti’s current global health research architecture, led and implemented by foreigners far removed from Haiti, demonstrates the sector’s “non-global” nature. Decolonization in Haiti must start by addressing the vast inequities that comprise this structure.

## Key Messages

- Global health work in Haiti is underpinned by structural racism and power hierarchies that perpetuate numerous inequities. In Haiti, many studies have been conducted by individuals from foreign research teams during periods of public health crises while giving few opportunities for Haitian community organizations to collaborate and participate in these interventions and failing to acknowledge the critical role of these organizations in facilitating their research.
- Global health research can be likened to a form of plunder, whereby those who are already powerful (e.g., researchers from high-income countries) are strengthened, while those located outside the reach of power (e.g., individuals and communities in low- and middle-income countries) are decimated.
- The successful decolonization of global health research in Haiti necessitates providing Haitian scholars with increased support and funding, more opportunities for training support and education, equitable partnerships and inclusion in research endeavors, and promotion in the development of research and publications from that research.

## INTRODUCTION

Recently, researchers have strongly emphasized the decolonization of global health—defined as “reversing the legacy of colonialism in health equity work.”^1^ The ongoing conversations and underlying momentum toward decolonizing global health have taken place primarily within high-income countries (HICs), with limited inclusion of individuals who are from or based in low- and middle-income countries (LMICs), where nearly all global health research occurs.

In countries such as Haiti, where injustice is dominant, global health functions under the guise of providing humanitarian services but operates as a different form of colonization from the Global North. Although there has been recent acknowledgment regarding the underlying racism and white power structures that perpetuate inequities within global health, interventions and efforts to address these inequities are limited. The current architecture of global health research demonstrates that global health is not global, as those leading and implementing research projects (often with robust funding) consist mainly of individuals from HICs, with limited projects being led by individuals and institutions in LMICs.^2^

We were compelled to write this commentary after collaborating on a systematic review in which we aimed to evaluate the state of public health and social science research in Haiti over the span of the last 2 decades. This review began in 2021 and is still in progress. In conducting this review, our research team noted that the majority of studies in Haiti were led by foreign research teams, primarily during moments of humanitarian crises. We realized there was a dearth of research focusing on the long-term sustainability, development, and evaluation of public health and social interventions in Haiti. Furthermore, the majority of studies that we identified failed to acknowledge the crucial role that Haitian community-based organizations and partners played in facilitating research for many foreign teams. Only a small number of studies provided acknowledgment to Haitian community-based organizations, and even fewer included Haitian collaborators as co-authors. These findings prompted our research team, which is composed of all Haitian female researchers, to further dissect the inadequacies in global health research and practice within Haiti. In this commentary, we provide a historical lens on colonization in Haiti and how it has contributed to the colonization of global health research within the Haitian context. Furthermore, we discuss whether the decolonization of global health is achievable and offer recommendations to promote collaborative partnerships in future global health research and practice.

## COLONIALIST FRAMEWORKS IN GLOBAL HEALTH IN HAITI

Global health work in Haiti, which mirrors that of many other LMICs, is underpinned by structural racism and power hierarchies that perpetuate numerous inequities. Historically known to be the poorest country in the Western hemisphere, Haiti has been crippled by a multitude of devastating natural disasters, a perilous political infrastructure, social violence, and extreme poverty.^3^^,^^4^ The unnerving social, political, and economic infrastructure of Haiti has fashioned the country’s former and current health experiences and evolution. In the past, colonial powers justified their conquests by asserting that they had an obligation to take over the land and culture of indigenous peoples with the argument that they were acting in the best interests of those whose lands and peoples they exploited.^5^ These colonialist ideas reflect what can be seen in global health work within present-day Haiti. A prime example of this is how Haiti has been named “The Republic of NGOs” because it has the largest number of nongovernmental organizations (NGOs) per capita in the world.^6^^,^^7^ Nearly 80% of Haiti’s basic health services are provided by the private sector through NGOs, with estimates indicating that more than 10,000 NGOs exist in the country, including a significant increase after the 2010 earthquake.^6^

Global health work in Haiti is underpinned by structural racism and power hierarchies that perpetuate numerous inequities.

Conflicting perspectives exist on whether the presence of NGOs and foreign researchers in Haiti benefits or harms the country. According to Guillaume et al., nonprofit organizations, such as Zanmi Lasante (Partners in Health), have increased the population’s access to health care and made tremendous strides in improving health outcomes for communities.^8^ Although many foreign entities arrive with the intention of helping, often they can function as a significant hindrance to the improvement of health, social, and economic outcomes for most Haitians. This was apparent after the disastrous 2010 earthquake when millions of dollars of donor funds were squandered and mismanaged due to ill-conceived and poorly executed NGO-led projects that did not benefit Haitians and worsened outcomes.^9^ We emphasize the dominant presence of NGOs because they have a strong influence on shaping global health research within Haiti.

In the context of global health research, the recurrent pattern is that researchers from HICs travel to Haiti, collect their data from vulnerable communities facing insurmountable circumstances, and are never to be seen or heard from again. This dynamic has been perpetual, in which researchers from foreign institutions collect data and serve interests that are far removed from and uncommitted to the local context.^10^ Consequently, global health research then becomes a form of plunder in strengthening those who are already powerful (e.g., researchers from HICs) while decimating those located outside the reach of power (e.g., communities in LMICs).^10^^–^^12^ Abimbola questions whether the daily and often unrecognized public health work done by nationals in LMICs within their own countries qualifies as global health or if global health solely applies to research conducted by a person with funds from HICs.^13^ As described by Beld, the current schematic that underpins global health research encourages foreign researchers to travel to countries and collect data without the local communities benefiting.^14^ This dynamic in global health has been normalized and heavily funded by private and public entities. As a result, many researchers fail to address critical global health issues or respect cultural values or indigenous health care methods, which may not align with their strict institutional protocols and Western ideologies. Instead, many research protocols focus on meeting institutional priorities and generating numbers rather than developing and strengthening community health systems. As stated in Cochran et al., an Alaska Native said, “Researchers are like mosquitoes; they suck your blood and leave.”^15^

## OVERVIEW OF LITERATURE REVIEW FINDINGS

Findings from our literature review revealed a tremendous influx of global health research in Haiti during periods of humanitarian crises, with little research being conducted after these marked periods. Among the included articles from our search (n=157), which spanned from 1979 to 2020, more than half (56.7%) of the studies that took place in Haiti occurred after the 2010 earthquake. During this time, the number of published articles on research based in Haiti doubled, spanning topics of disaster relief, emergency medicine, infectious diseases, intimate partner violence, sexual and reproductive health, and other significant public health issues. The majority of studies were conducted by authors based in the United States (n=117 [74.5%]), followed by countries outside of the United States (n=27) and Haiti (n=13).

Our literature review revealed a tremendous influx of global health research conducted in Haiti during humanitarian crises, with little research done afterwards.

A topic that gained popularity among researchers during this period was the 2010 cholera outbreak, which resulted in researchers from Western countries leading studies on cholera surveillance, disease modeling, qualitative field studies with communities, and implementation science research. However, few of these studies were led by Haitian authors. Furthermore, many of the studies failed to acknowledge the critical role of the Haitian Ministry of Health in establishing a national plan for cholera elimination, despite the U.S. Centers for Disease Control and Prevention having highlighted the response as among the best coordinated and documented responses to a cholera outbreak in modern public health.^8^^,^^16^ This action was spearheaded by the Haitian government and international partners, including the U.S. Centers for Disease Control and Prevention and Partners in Health.

After Hurricane Matthew in 2016, there was another substantial increase in research studies being conducted in Haiti (n=16 [10.2%]). This was followed by a marked decline in publications in 2017 (n=7 [4.5%]) until 2018 during a period of political upheaval (n=15 [9.6%]). These findings indicate a trend of increases in global health research in Haiti during periods of humanitarian crises, followed by pronounced reductions in research afterward. Consequently, due to research being solely conducted in response to crises, it becomes clear why there has been a severe dearth of global health research and initiatives in Haiti that have resulted in the development of sustainable interventions that can benefit communities at large.^8^^,^^16^

## IS GLOBAL HEALTH HELPING OR CAUSING HARM?

The relationship between Haiti and foreign institutions in HICs that conduct global health work is complex and multifaceted. Haiti continues to fall victim to economic and political strife, foreign interventions, and natural disasters, thus abdicating itself to a lamentable health care infrastructure. As a result, there is a belief among some Haitians themselves that foreign aid is needed through partnerships to help the country in responding to health issues.^17^ However, it is imperative that equity underpins these partnerships. While global health has been framed by many researchers as a call for equity and justice, it is clear that it solely serves the needs of primarily white and foreign researchers and institutions, thus upholding colonial legacies.^18^

The decolonization of global health mandates the removal of all forms of white supremacy within countries, between countries, and at the global level.^19^ It requires researchers to conduct equitable research that prioritizes the needs and interests of target communities, with the goal of developing solutions to complex public health problems. Khan et al. detailed the steps needed to achieve reform in global health and to mitigate existing power structures while promoting equity.^20^ However, if the decolonization of global health is to transition from theory into actual practice, one must question how global health will continue to function if the existing power structures are removed. It could be argued that perhaps global health would not function as we know it today given the power structures embedded in colonialism and racism at the core of global health. These power structures largely benefit from the perpetual marginalization of local communities, which results in the lack of sustainable change. The decimation of these power structures would require the removal of the privileges that global health in its current form provides foreign researchers. With these considerations in mind, our research team questioned: Does the decolonization of global health actually exist, or is it all just rhetoric with no intention of informed action?

## RECOMMENDATIONS FOR DECOLONIZING GLOBAL HEALTH IN HAITI

Global health research in Haiti maintains the gap in sustainability between work that is produced and the long-term change that is needed. It is imperative for research to produce knowledge and contribute to the positive changes that communities need and envision for themselves.^21^^,^^22^ Data from research conducted in Haiti must be used to address relevant health issues and provide attainable recommendations that are adequate, respectful, culturally appropriate, and that honor the Haitian people. There is an urgent need for a paradigm shift to decolonize global health initiatives in Haiti. The authors recommend the following actions to address these issues and proactively mitigate the effect of colonization on global health research in the Haitian community: (1) increase support and funding of Haitian scholars, (2) provide opportunities for training and continued education, and (3) promote the research and publication of Haitian scholars and collaborators.

Data from research conducted in Haiti must be used to address relevant health issues and provide attainable recommendations that are adequate, respectful, and culturally appropriate.

### Increase Support and Funding of Haitian Scholars

One of the biggest challenges Haitian scholars face is a lack of funding. The socioeconomic situation in Haiti does not allow the availability of grants and fellowships to support the Haitian scientific community. As in many LMICs, the government in Haiti does not allocate substantial resources toward research and capacity-strengthening for academic institutions. This leaves universities in Haiti with no choice but to collaborate with institutions in HICs for funding.^23^ This structural issue must be addressed, and priority must be placed on providing funding for public academic institutions in support of research efforts. International funding mechanisms should also be reinforced to support the development of Haitian scholars. The lack of priority placed on research in Haiti often results in students not being exposed to research careers. For individuals who demonstrate interest in a research career, options within Haiti are severely limited. Increasing the availability of national and international grants and fellowships for researchers in Haiti can increase the production of scientific studies relevant to the needs of the Haitian community.

Global health research in Haiti should aim to provide the community with robust scientific knowledge and evidence for adequate, sustainable, and inclusive global health development. Haitian scholars, in addition to being qualified, possess cultural and contextual knowledge that allows them to better understand the aspects related to Haitian mores and customs. From this perspective, collaborative research knowledge should be encouraged to include more Haitian scholars in research endeavors. Unfortunately, in many instances, researchers in LMICs have limited opportunities for continuity beyond project funding life cycles.^23^ Scientific and academic institutions, along with NGOs and partners conducting research and public health work in Haiti, should recruit more Haitian scholars. Policies should be established that limit the number of foreign institutions conducting work that can be performed by Haitian locals.

### Provide Opportunities for Training and Continued Education

The deficiency of Haitian research production is linked in part to the insufficiency of the research laboratories and programs in the country. The dearth of graduate programs in Haitian universities contributes to the lack of research skills. Higher education in Haiti consists of the state university (Université d’État d’Haïti) and a few private universities. Given the lack of public universities, the cost of attending private universities, and the concentration of universities in the western part of the country, especially in Port-au-Prince, access to higher education is extremely restricted. Aside from the lack of universities and programs, the lack of satisfactory libraries and qualified professors also reinforces the challenge. Rebuilding efforts for higher education in Haiti should address these prerogative issues for optimal efficiency of the Haitian scientific system. Haitian universities must be endowed with better resources and graduate programs (master’s and doctoral) that respond to international and scientific standards. Support for training and continued education should be provided by local institutions and organizations, along with international entities, to strengthen the research community.

Haitian universities must be endowed with better resources and graduate programs that respond to international and scientific standards.

### Promote the Research and Publication of Haitian Scholars

The decolonization of global health research should start in the classroom by allowing future professionals to be trained with materials that are adequate, equitable, cross-cultural, and devoid of Western white supremacy. Scientific research led by Haitian and other Black scholars should be promoted within the academic community to increase the awareness of research and strengthen the decolonization movement through organizing symposiums, scientific forums, and webinars. The university professors can also contribute to the promotion of Haitian publications by including Haitian scholars in their lectures and encouraging the student to study Haitian authors. Foreign researchers who conduct global health research in Haiti must properly acknowledge Haitian collaborators and include them as co-authors and lead authors in publications. First and senior authors are often collaborators in HICs.^23^ Haitian researchers who are instrumental to the success of projects lack opportunities to contribute to and lead publications, thus giving rise to poor career trajectories.

It is also important to highlight the need for more decolonization of global health research advocates, including committed models, adequate mentoring, and Haitian scientists that are working in foreign countries. Political turmoil and socioeconomic problems coerce a significant proportion of graduates of the education system, educated at the expense of Haitian taxpayers, to emigrate to North America and Europe.^24^ Others have benefited from an international scholarship for further education in another country and decided to stay there to seek better career opportunities. We recommend strengthening or reinforcing socioeconomic and political stability and creating better professional opportunities in Haiti to stop the mass professional migration and encourage Haitian researchers from the diaspora to return home and strengthen the research community.

## CONCLUSIONS

This commentary highlights the impact of colonization on global health research in the Haitian context. Specifically, it addresses the need for research focusing on the long-term sustainability of global health interventions in Haiti and the engagement of Haitian research teams and community-based organizations in the production of scientific knowledge. Researchers from HICs must aim to understand the culturally specific needs of Haitian individuals and work diligently to partner with Haitian researchers on-site and include them in funded grants and publications. In 2021, the senior author of this commentary (GN) established a research committee with the co-authors under the Haitian American Professionals Coalition.^25^ Our group aims to develop and support research endeavors within Haiti and the Haitian diaspora, along with providing mentorship to Haitian scholars. We propose that this initiative composed of Haitian researchers can have a substantial impact in strengthening research and scholarship in Haiti. Although it cannot be refuted that Haiti has benefited from many endeavors conducted by foreign academics and institutions, the long-term impact may not be fully realized without the ongoing development of Haitian scholars. In the same manner that giving a person a fish does not provide the same result as teaching them to fish, without specific actions to promote Haitian scholarship and research to create self-sustaining programs, the health and well-being of the Haitian people will continue to be reliant on foreign interventions that often fail to adequately address the needs of the Haitian people.

## Notes

**Peer Reviewed**

**First published online:** June 13, 2023.
